# The Neural Persistence of Memory: Retention Begins While You're Still Awake

**DOI:** 10.1371/journal.pbio.0040116

**Published:** 2006-03-28

**Authors:** Liza Gross

There's some unwritten law of stadium parking that says after any event some fraction of hapless souls must perform an embarrassing reenactment of *Dude, Where's My Car?* It might seem like a simple thing to remember until you consider that the brain must often process and retain new memories while simultaneously tending to several unrelated cognitive tasks. Though it's not exactly clear how the brain processes a recent memory, evidence suggests that a good nap during an event might prevent parking mishaps. Many studies have shown that brain regions activated while learning a task are reactivated during sleep, suggesting that this “offline” processing facilitates memory retention. But when does the memory consolidation process begin?

Studies in rodents and monkeys have shown that the same neuron ensembles activated during the practice phase of a task continue to be activated for several minutes immediately after exposure to a new task. This suggests that delayed activation in the brain represents a step in the memory storage process.

In a new study, Phillippe Peigneux, Pierre Orban, Pierre Maquet, and their colleagues used functional magnetic resonance imaging to test this possibility and probe the fate of recent memories in the human brain. The researchers asked individuals to perform two separate tasks associated with different memory systems, and found that regional brain activity associated with learning a task persisted and evolved while participants completed an unrelated task. The learning-dependent changes in regional brain activity they observed while individuals were awake echoed those seen during sleep. This offline activity may act as a placeholder, maintaining newly acquired information until it gets transferred to long-term storage during the memory consolidation process.

The researchers chose spatial and procedural tasks that are known to induce post-training brain activity in learning-related sectors during sleep. Each task engages a different brain sector—the spatial task depends on the hippocampus while the procedural task relies on cortical and subcortical regions—allowing the researchers to distinguish each task's post-training brain activity from activity associated with practicing a different task.

For the spatial task, participants navigated a path through virtual space; for the procedural task, they indicated under which of four position markers a dot appeared by rapidly pressing a keystroke. For the unrelated “oddball task,” participants lay in the scanner and mentally counted the deviant sounds embedded in a monotonous soundtrack. These oddball sessions occurred immediately before a task—providing baseline brain activity—immediately after a 30-minute training session, and again after a 30-minute rest period. A short behavioral test followed the last oddball session, then participants were scanned a fourth time while performing their task to identify brain regions associated with each task. Two weeks later, individuals were tested on the alternate task, so the researchers could compare post-training modulated brain activity associated with each task.

Brain responses to the oddball task were significantly higher immediately after training on the spatial task than they were in the pre-training session. Delayed post-training activation (after the break) also remained significantly higher in the hippocampus and other brain regions associated with spatial navigation. The pattern for the procedural task was similar but followed a different time course. Brain activity in cortical and subcortical regions associated with task performance decreased immediately after training but then showed a delayed increase, above pre-training levels, in learning-related brain sectors.

For both tasks, modulated offline activity showed a tighter coupling with other brain regions associated with learning each task following the training period; this coupling occurred immediately after training for the spatial task and after a 45-minute delay for the procedural task. The researchers went on to relate these post-training, task-dependent, regionally specific changes to post-training performance.

The relationship between behavioral performance and functionally significant brain activity changes suggests that this offline activity plays a role in maintaining and processing newly acquired memories. Moreover, the researchers argue, these neural correlates of memory maintenance—persistent and reorganized neural activity that occurs while you're alert and tending to other matters—operate in different brain regions at different times to process distinct types of memories. It remains to be seen whether persistent neural traces continue after memories are consolidated. So you may not need that nap to remember where you parked your car after all—but it wouldn't hurt to jot down the location, just in case.

## 

**Figure:**
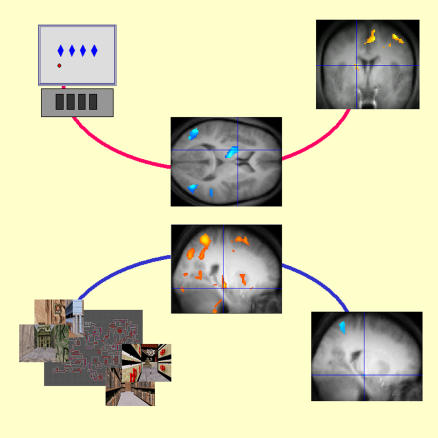
After participants learned to perform either a procedural or a spatial task, learning-dependent, regional brain activity persisted and evolved with time, suggesting the neural integration of recent memories.

